# Rapid Hepatobiliary Excretion of Micelle-Encapsulated/Radiolabeled Upconverting Nanoparticles as an Integrated Form

**DOI:** 10.1038/srep15685

**Published:** 2015-10-23

**Authors:** Hyo Jung Seo, Sang Hwan Nam, Hyung-Jun Im, Ji-yong Park, Ji Youn Lee, Byeongjun Yoo, Yun-Sang Lee, Jae Min Jeong, Taeghwan Hyeon, Ji Who Kim, Jae Sung Lee, In-Jin Jang, Joo-Youn Cho, Do Won Hwang, Yung Doug Suh, Dong Soo Lee

**Affiliations:** 1 Department of Molecular Medicine and Biopharmaceutical Sciences, Graduate School of Convergence Science and Technology, Seoul National University, Seoul, Korea; 2 Department of Clinical Pharmacology and Therapeutics, Seoul National University College of Medicine, Seoul, Korea; 3 Department of Nuclear medicine, Seoul National University College of Medicine,Seoul, Korea; 4 Laboratory for Advanced Molecular Probing (LAMP), Research Center for Convergence Nanotechnology, Korea Research Institute of Chemical Technology, Daejeon, Korea; 5 Center for Nanoparticle Research, Institute for Basic Science (IBS), Seoul, Korea; 6 School of Chemical and Biological Engineering, Seoul National University, Seoul, Korea; 7 School of Chemical Engineering, Sungkyunkwan University, Suwon, Korea

## Abstract

In the field of nanomedicine, long term accumulation of nanoparticles (NPs) in the mononuclear phagocyte system (MPS) such as liver is the major hurdle in clinical translation. On the other hand, NPs could be excreted via hepatobiliary excretion pathway without overt tissue toxicity. Therefore, it is critical to develop NPs that show favorable excretion property. Herein, we demonstrated that micelle encapsulated ^64^Cu-labeled upconverting nanoparticles (micelle encapsulated ^64^Cu-NOTA-UCNPs) showed substantial hepatobiliary excretion by *in vivo* positron emission tomography (PET) and also upconversion luminescence imaging (ULI). *Ex vivo* biodistribution study reinforced the imaging results by showing clearance of 84% of initial hepatic uptake in 72 hours. Hepatobiliary excretion of the UCNPs was also verified by transmission electron microscopy (TEM) examination. Micelle encapsulated ^64^Cu-NOTA-UCNPs could be an optimal bimodal imaging agent owing to quantifiability of ^64^Cu, ability of *in vivo*/*ex vivo* ULI and good hepatobiliary excretion property.

Although nanoparticles (NPs) have a huge potential as both diagnostic imaging and therapeutic agents, a majority of attempt for clinical translation has been blocked by unsolved biocompatibility issues. Indeed, delayed excretion and non-biodegradability of NPs have caused overt toxicity and tissue damage^1^,^2^,^3^,^4^,^5^. Many reports demonstrated that NPs are persistently taken by the mononuclear phagocyte system (MPS) and induce tissue toxicity in liver and spleen^3^,^6^. One the other hand, by modifying surface characteristics, NPs could be excreted via hepatobiliary excretion pathway without overt tissue toxicity^7^,^8^. Therefore, it is critical to develop the biocompatible NPs that show favorable excretion property to minimize toxicity *in vivo*^9^.

Micelle encapsulation method for hydrophilization of nanomaterials was first described by Dubertret *et al.*^10^ and further refined by several groups^11^,^12^,^13^. Recently, our group integrated the method and proposed one-step method under mild conditions to preserve ligand integrity to make functionally active multi-specific nanoparticles^14^. However, biodistribution and excretion pattern of micelle encapsulated nanoparticle was not well evaluated until now. Especially, whether the integral nanoparticle or disintegrated parts are excreted and whether even the post-administration aggregation influences the biodistribution pattern have not been well elucidated.

Upconverting nanoparticles (UCNPs) have recently received interest for their *in vivo* imaging capability that can emit visual light upon absorption of long wavelength near-infrared (NIR) photons. Indeed, upconversion luminescence imaging (ULI) may provide the advantages of autofluorescence-free nature^15^, good penetration depth, and low photodamage in cells^16^. Also, to enhance water solubility and biocompatibility, surface coating of UCNP using phospholipid has been reported^17^. Positron emission tomography (PET) has been considered as useful imaging modality for biodistribution study with high sensitivity, depth penetration and capability of quantification^18^.

In the present study, we demonstrated the micelle-encapsulated labeling method of a bimodal imaging agent, and that the biodistribution and excretion of these NPs are favorable as the platform for *in vivo* use using *in vivo* PET and ULI. We reinforced imaging results by *ex vivo* biodistribution study and feces evaluation, and finally explored the mechanism of hepatobiliary excretion by transmission electron microscopy (TEM) of the liver.

## Results

### Synthesis of micelle encapsulated ^64^Cu-NOTA-UCNPs

**Figure 1:**
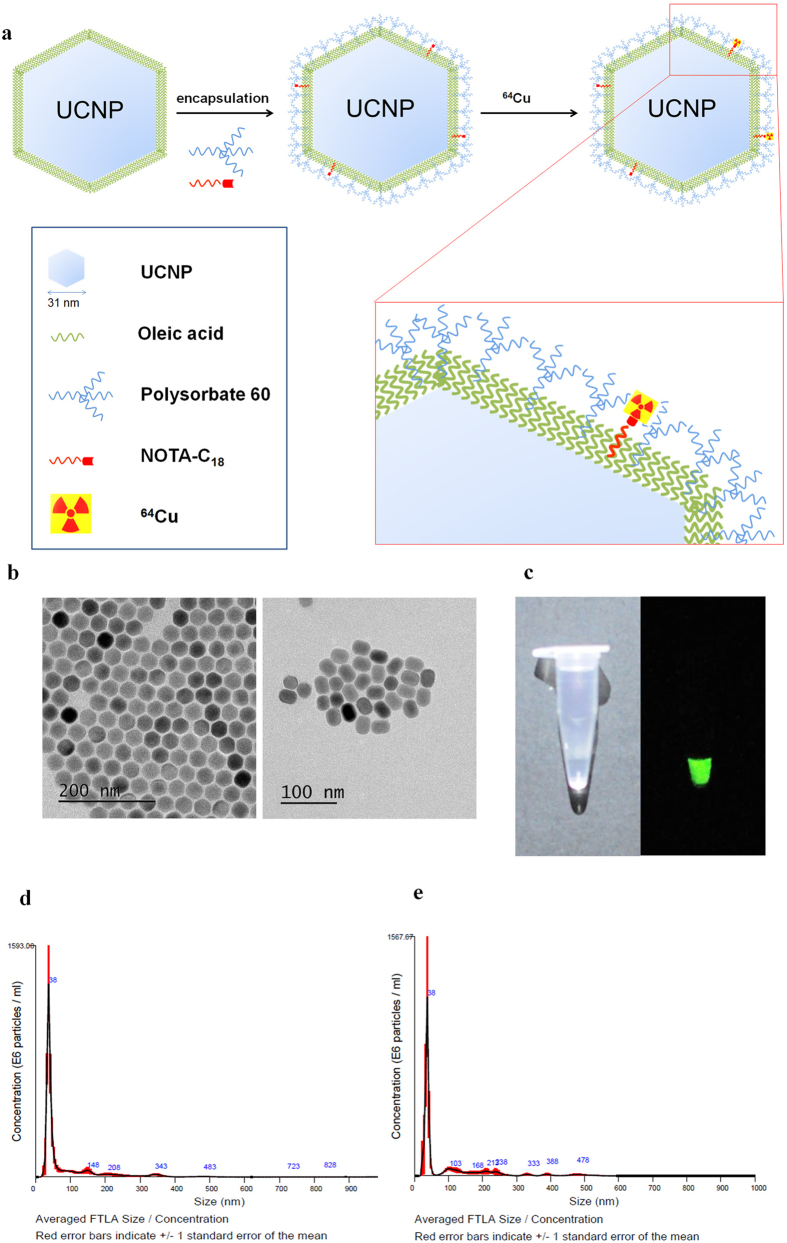
Synthesis and characterization of micelle encapsulated ^64^Cu-NOTA-UCNP. (**a)** Polysorbate 60 was used to transfer the hydrophobic UCNPs into aqueous phase and NOTA-C_18_ was used for the radiolabeling. Then, ^64^Cu was added and labeled with NOTA-UCNPs. Enlarged configuration of micelle encapsulated ^64^Cu-NOTA-UCNP was included. Compounds including UCNP, polysorbate 60, NOTA-C_18_, oleic acid and ^64^Cu are listed in the blue box. (**b**) TEM image of UCNPs before (left) and after (right) micelle encapsulation (**c**), Upconversion luminescence image of UCNPs under 980 nm laser excitation. (**d,e)** Size of micelle encapsulated NOTA-UCNPs (**d**) and micelle encapsulated ^64^Cu-NOTA-UCNPs (**e**) measured by nanoparticle tracking analysis (NTA) method showing sharp peak which indicates uniform size of the nanoparticles. Size of micelle encapsulated ^64^Cu-NOTA-UCNPs showed similar size with the micelle encapsulated NOTA-UCNPs.

The entire labeling method of micelle encapsulated ^64^Cu-NOTA-UCNPs was illustrated in Fig. 1a. UCNPs showed hexagonal shape on TEM, and were not changed after micelle encapsulation (Fig. 1b). The micelle encapsulated UCNPs in phosphate buffer solution (PBS) showed visible green light by 980-nm laser excitation (Fig. 1c). Using micelle encapsulation method, the radiochemical purity of micelle encapsulated ^64^Cu-NOTA-UCNPs was higher than 99%. The mode size of micelle encapsulated UCNPs, and micelle encapsulated ^64^Cu-NOTA-UCNPs were 34.1 ± 6.4 nm and 34.4 ± 2.6 nm, respectively (Fig. 1d,e). The zeta potential of micelle encapsulated UCNPs and micelle encapsulated ^64^Cu-NOTA-UCNPs were −27.1 ± 5.43 mV and −6.63 ± 6.31 mV, respectively.

### *In vivo* PET Imaging of micelle encapsulated ^64^Cu-NOTA-UCNPs

**Figure 2:**
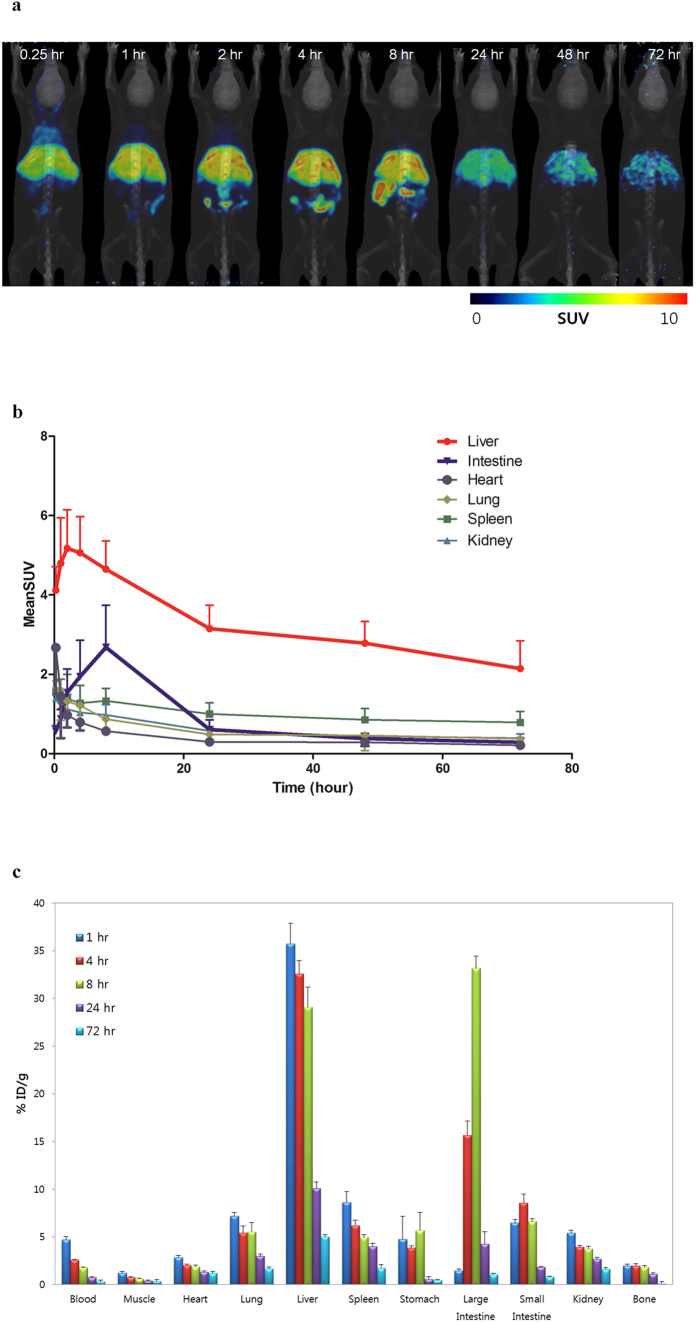
*In vivo* whole body microPET imaging and biodistribution study in micelle encapsulated ^64^Cu-NOTA-UCNPs-injected mice. (**a**) Decay-corrected MIP (Maximum Intensity Projection) images at different time points after intravenous injection of micelle encapsulated ^64^Cu-NOTA-UCNPs. Hepatic uptake was increased until 2 hours delay image, and then decreased serially. The blood pool activity was visualized in heart, lungs and both kidneys at 0.25 hour image and disappeared at 1 hour delay image. Segmental uptake in the intestine was noted after 1 hour. The linear intestinal uptake was seen until 8 hours and was minimal at 24 hours after injection. (**b**) Quantitative analysis of *in vivo* PET image in mice after intravenous injection of micelle encapsulated ^64^Cu-NOTA-UCNPs. The data corresponded to visual analysis of the PET images. (**c**) *Ex vivo* biodistribution in mice after intravenous injection of micelle encapsulated ^64^Cu-NOTA-UCNPs. Data (n = 3) were expressed as mean ± SD, indicating the percentage administered activity (injected dose) total gram of tissue (% ID/g). The uptake of liver markedly decreased at 24 hours. Radioactivity of intestine increased until 8 hours and decreased at 24 hours. MeanSUV in (**b**) represents mean standardized uptake value. Error bars in (**b**,**c**) correspond one standard deviation.

*In vivo* PET images were acquired serially after intravenous administration of micelle encapsulated ^64^Cu-NOTA-UCNPs via tail vein injection (Fig. 2a). Initially, tracer uptake was shown primarily in the liver and the lungs at 0.25 hour PET image. Hepatic uptake persisted until 8 hours. At 1 hour, uptake in the intestine was observed and extended downstream along the intestine at 2, 4 and 8 hours. Interestingly, uptakes in the liver markedly decreased at 24 hours compared to 8 hours, and this decreased further until 72 hours (Fig. 2a). The serial PET images indicated that micelle encapsulated ^64^Cu-NOTA-UCNPs were excreted by hepatobiliary system on visual inspection. The amount of radioactivity in each organ which was quantified by region of interest analysis was consistent with the visual findings (Fig. 2b). The *ex vivo* biodistribution data were also consistent with quantified PET data that the concentration of micelle encapsulated ^64^Cu-NOTA-UCNPs in the liver decreased further until 72 hours. 84% of the initial hepatic uptake was excreted at 72 hours. Meanwhile, the concentration of micelle encapsulated ^64^Cu-NOTA-UCNPs in the intestine increased in small intestine first and then large intestine, and decreased at 24 hours due to the expulsion of feces (Fig. 2c).

### Free ^64^Cu or ^64^Cu-NOTA-C_18_ are not likely detached from micelle encapsulated ^64^Cu-NOTA-UCNPs *in vivo*

To examine the possibility of the detachment of free ^64^Cu or ^64^Cu-NOTA-alkyl chain (^64^Cu-NOTA-C_18_) from the injected micelle encapsulated ^64^Cu-NOTA-UCNPs, PET images using free ^64^Cu (n = 3) or ^64^Cu-NOTA-C_18_ (n = 3) were acquired. ^64^Cu or ^64^Cu-NOTA-C_18_ showed different biodistribution from micelle encapsulated ^64^Cu-NOTA-UCNPs in *in vivo* PET image (Supplementary Fig. 1a). ^64^Cu-NOTA-C_18_ showed initial high bladder activity at 0.25 hr image, indicating rapid renal excretion which is minimal in micelle encapsulated ^64^Cu-NOTA-UCNPs. Absence of initial bladder uptake of the micelle encapsulated ^64^Cu-NOTA-UCNPs support that ^64^Cu-NOTA-C_18_ was not detached from them which is understandable considering the size of the UCNPs^19^. Both micelle encapsulated ^64^Cu-NOTA-UCNPs and ^64^Cu-NOTA-C_18_ showed initial higher hepatic uptake and faster excretion than free ^64^Cu. Also, free ^64^Cu activity in heart persisted until 24 hours unlike ^64^Cu-NOTA-C_18_ and micelle encapsulated ^64^Cu-NOTA-UCNPs, probably due to recirculation of the copper ion^20^ (Supplementary Fig. 1b). Free ^64^Cu or ^64^Cu-NOTA-C_18_ were not likely detached from the micelle encapsulated ^64^Cu-NOTA-UCNPs *in vivo*. In one recent report, ^111^In-DOTA was detached *in vivo* from the ^111^In-DOTA-amphiphile encapsulated Au particles and excreted via kidneys^21^, which is in contrast to our findings. What determines *in vivo* detachment of chelators from the amphiphiles, i.e., nature of the core, nature of the amphiphile, or the chemical bond between chelator and amphiphile, is to be understood.

### *In vivo* luminescence imaging shows pattern of hepatobiliary excretion

**Figure 3:**
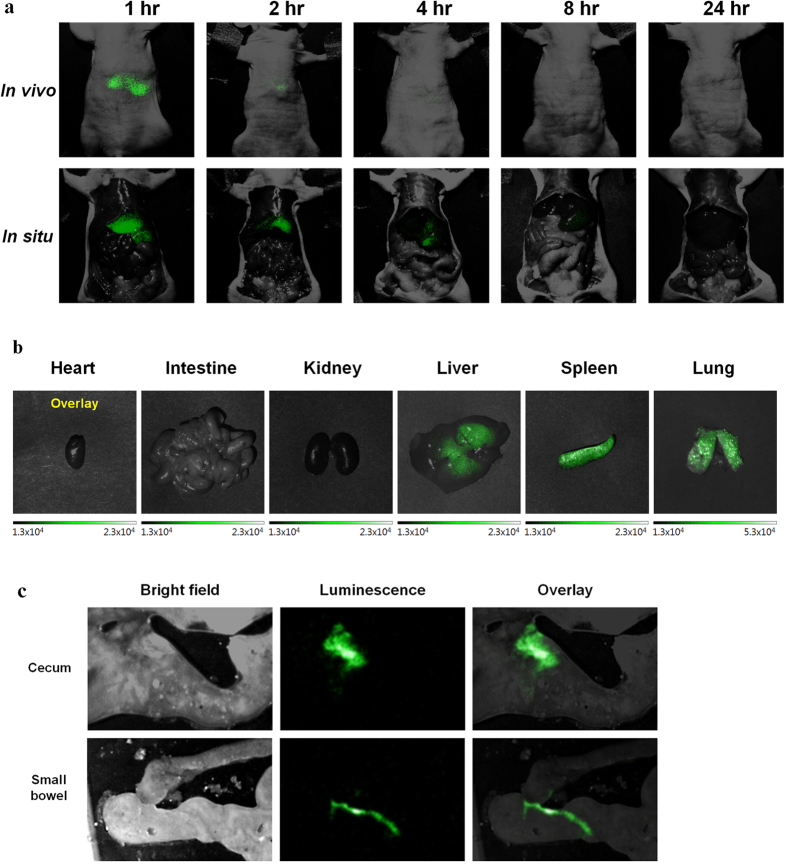
*In vivo* upconversion luminescence images in nude mice. (**a**) *In vivo* and *in situ* images at different time points. Upconversion luminescence signal was visualized in the liver area at 1 hour after the injection and disappeared in *in vivo* image. In *in situ* images, upconversion luminescence signal in the liver was shown and decreased until 8 hours delay images and it was not seen on 24 hours delay image. (**b**) *Ex vivo* upconversion luminescence image at 1 hour image showed positive signals in the lungs, liver and spleen. (**c**) Four hours after the injection, multifocal luminescence signals could be seen along the lumen of the intestine. All images were acquired under the same experimental condition.

Luminescence imaging of UCNPs was done at serial time points using in-house built apparatus. In *in vivo* and *in situ* luminescence imaging, initial liver uptake at 1 hour decreased faster at further time points than in PET (Fig. 3a). This apparent rapid disappearance of luminescence signal was probably due to limited depth-penetrance of luminescence signals emitted from the UCNPs in the hepatic porta and the intestines. At 1 hour *ex vivo* image, liver, spleen and lungs showed signals reminding consistent *ex vivo* biodistribution results (Fig. 3b). The intestinal luminescence by UCNPs was clearly seen after exposure of intestinal contents (Fig. 3c). This intestinal uptake was prominent until 8 hours delay, which was in accordance with the *in vivo* PET and *ex vivo* biodistribution studies showing hepatobiliary excretion. ULI imaging *in situ* after animal sacrifice complied well with PET images and their quantification results as well as *ex vivo* biodistribution data.

### Integral ^64^Cu-NOTA-UNCPs are excreted to feces and urine

**Figure 4:**
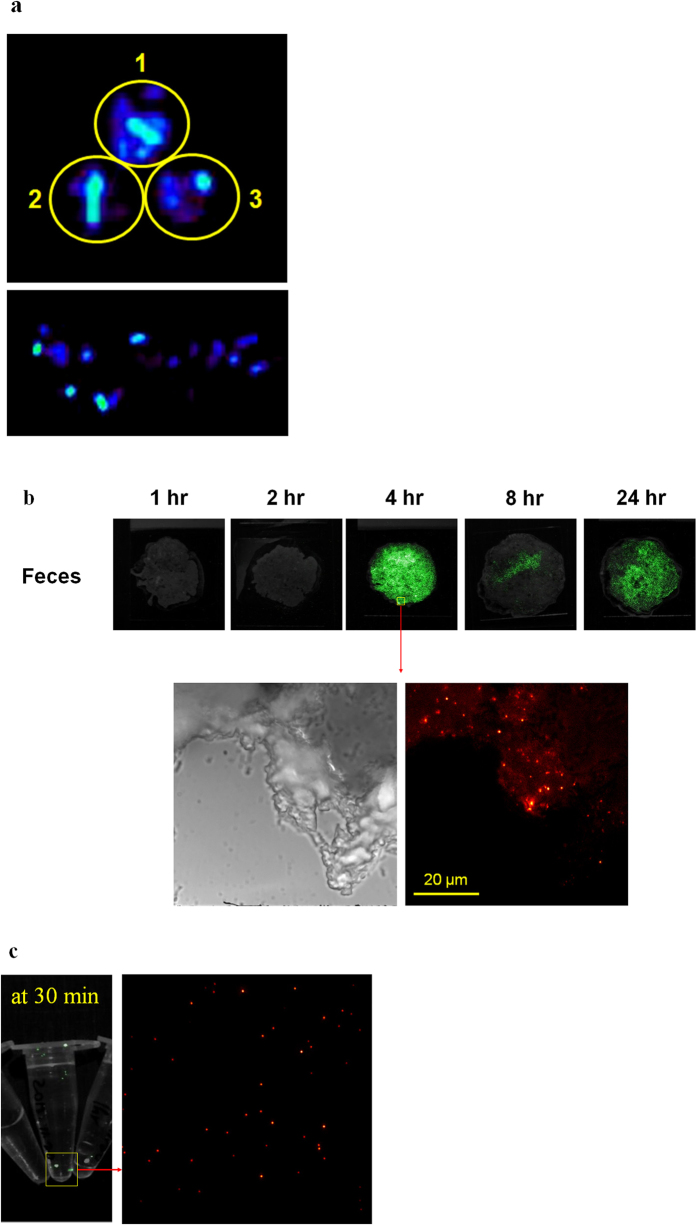
Evidences of hepatobiliary and urinary excretion using multimodal imaging and methods. (**a**) PET image showed increased uptake in collected feces. All feces (n = 3) showed heterogeneously increased uptake. Yellow circles with numbers indicate that the feces were from different mice. (**b**) Upconversion luminescence images of feces at serial time points. Upconversion luminescence signal was observed in all collected feces after 4 hour post-injection. Microscopic image shows multiple and scattered UCNPs. (**c**) Upconversion luminescence imaging (ULI) of collected urine at 30 minutes after ^64^Cu-NOTA-UNCP injection. Several spots with luminescence were observed inside of tube. Enlarged image shows multiple UCNPs.

For further verification of the hepatobiliary excretion of the micelle encapsulated ^64^Cu-NOTA-UNCPs, we collected feces and urine. Percentage of injected dose (%ID) in all of the collected feces and urine were calculated based on gamma scintillation analysis and the values were 40.9 ± 3.3, 1.1 ± 0.6%, respectively until 24 hour. Intense PET and upconversion luminescence signals were observed in most of the feces (Fig. 4a,b). On microscopic ULI, multiple scattered UCNP particles were found in the feces by their luminescence (Fig. 4b). Although in very small amount, urinary excretion of UCNP was confirmed by luminescence images, showing that spotty signal (Fig. 4c). We now know that integral ^64^Cu-NOTA-UNCPs were excreted mainly by feces and also by urine in small amount. It seems that the amount in urine was too small to be visualized in *in vivo* PET imaging (Fig. 2a, Supplementary Fig. 1a). There were several reports showing urinary excretion of nanoparticles with larger size than the pore of the glomerulus, however, the mechanism is not clear now^22^.

### Biliary excretion of UCNPs is revealed by TEM

**Figure 5:**
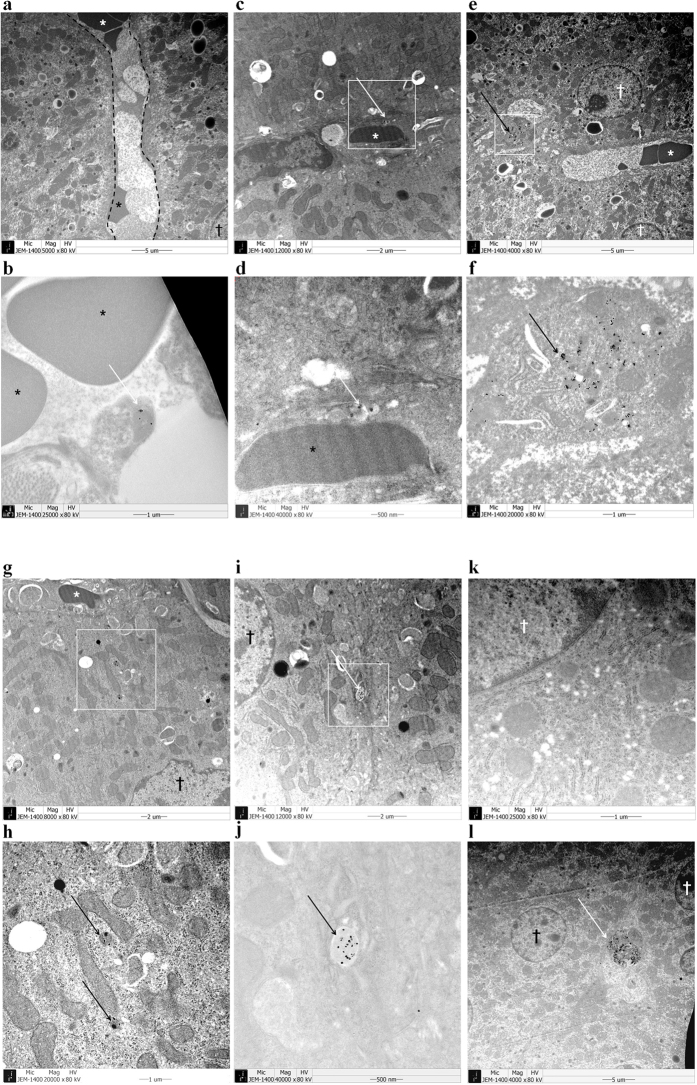
Observation of hepatobiliary excretion on TEM study. (**a**) Structure of hepatocytes and sinusoid is demonstrated. Black fenestrated lines are delineating sinusoid space. At 1 hour after injection of UCNPs, UCNPs were observed inside of sinusoid (**b**) and in the space of Disse (**c**,**d**) (**d**) = magnified image of white box area in (**c**). Multiple UCNPs were found in the intracellular space of Kupffer cell which is lying on sinusoid lining (**e**,**f**) (**f**) = magnified image of white box area in (**e**). UCNPs were found in cytoplasm of hepatocyte in vesicular structure (**g**,**h**) (**h**) = magnified image of white box area in (**g**). UCNPs were found inside the bile canaliculi (**i**,**j**) (**j**) = magnified image of white box area in (**i)**. At 24 hours after injection of UCNPs, UCNP was not seen in hepatocyte (**k**) but seen in Kupffer cell (**l**). Arrows indicate UCNPs. Asterisks (*) indicate red blood cells. Crosses (†) indicate nuclei of hepatocytes.

**Figure 6:**
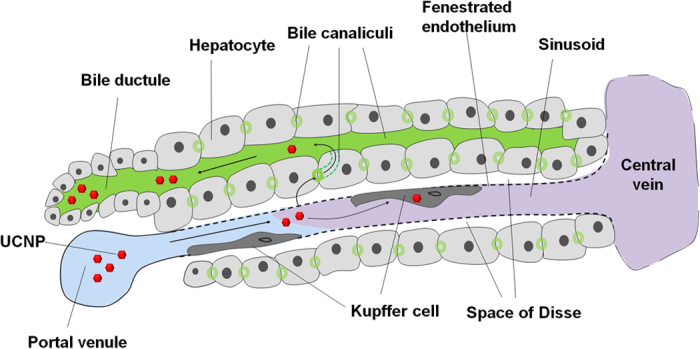
Schematic presentation of the kinetics of UCNPs in the liver tissue. Injected UCNPs reach sinusoid, which is a fenestrated capillary, via portal venule. In sinusoid, UCNPs can be phagocytosed by Kupffer cells or filtered out to space of Disse and endocytosed by hepatocytes. Endocytosed UCNPs to hepatocytes can go to biliary canaliculi and are excreted via biliary excretion pathway. This figure was drawn by Do Won Hwang and Hyung-Jun Im.

For mechanistic understanding of hepatobiliary excretion, TEM study was done using liver section of mice sacrificed at 1 hour (n = 3) and 24 hours (n = 3) after injection of micelle encapsulated ^64^Cu-NOTA-UCNPs. Normal structure of sinusoid and hepatocyte is demonstrated in Fig. 1a. At 1 hour, UCNPs was observed inside of sinusoid (Fig. 5b), space of Disse (Fig. 5c,d), Kupffer cells (Fig. 5e,f) and hepatocytes (Fig. 5g,h). Inside the hepatocytes, UCNPs are localized in cytoplasmic vesicles and some of the vesicles contained multiple UCNPs (Fig. 5g,h). Also, UCNPs were found in bile canaliculi which is an initial pathway of bile excretion (Fig. 5i,j). At 24 hours after injection, interestingly, UCNPs were observed in Kupffer cells but not inside of hepatocytes (Fig. 5k,l). These findings suggest that injected UCNPs were endocytosed by either hepatocytes or Kupffer cells in the liver and UCNPs in the hepatocytes were excreted via biliary excretion pathway. On the contrary, UCNPs in the Kupffer cells seem to remain inside these cells until 24 hours after injection (Fig. 6). The very small amounts of UCNPs on nanoparticle tracking analysis measurements with larger size indicating aggregates might explain the activity of UCNP aggregates taken up by the Kupffer cells (Figs 1e and 5l).

## Discussion

As in our *ex vivo* biodistribution results, over 80% clearance from initial liver uptake within 72 hours is quite substantial compared to previous reports showing hepatobiliary excretion of NPs^7^,^8^,^23^. Generally, nanoparticles with larger size than glomerulus pore initially retained in liver or spleen after intravenous administration^24^. After the NPs have reached to the liver, the NPs were considered to be endocytosed mainly by Kupffer cells or possibly by hepatocytes. NPs endocytosed by hepatocytes will enhance hepatobiliary excretion^19^. To be endocytosed by hepatocyte, size of NP should have been less than 100 nm because the size of fenestration of endothelium of the liver is around 100 nm^24^. Also, size around 50 nm is optimal to maximize the rate of endocytosis in several types of NPs^25^. Thus size of NP of the present study (34.4 nm) might have been optimal for hepatobiliary excretion. And/or micelle encapsulation of the nanoparticle should have facilitated hepatobiliary excretion. There were several earlier reports that showed hepatobiliary excretion of liposome^26^ and micelles^27^,^28^. In accordance with the reports, ^64^Cu-NOTA-C_18_, which would have formed micelles in the aqueous solvent, also showed substantial hepatobiliary excretion after initial urinary excretion. We suggest that micelle encapsulated ^64^Cu-NOTA-UNCPs are notified as micelles to the liver thus, more prone to be taken up by hepatocyte rather than Kupffer cell. Also, micelle encapsulation could lower the protein adsorption to NPs, thus lower the chance of phagocytosis by Kupffer cells^29^,^30^. Obviously, PEGylation and low surface negative charge of micelle encapsulated ^64^Cu-NOTA-UCNPs were necessary, though not sufficient, for substantial hepatobiliary excretion of the NPs. PEGylation and low surface charge of NPs are also known to reduce protein adsorption in serum and phagocytosis^19^,^31^. Accordingly, the rate of endocytosis of the NPs to hepatocytes could increase.

We found that 16% of the initial hepatic uptake of the micelle encapsulated ^64^Cu-NOTA-UNCPs remained in the liver at 72 hours after the injection. The remained NPs were probably to have been phagocytosed by Kupffer cells and thus not likely to be excreted via hepatobiliary excretion even after 72 hours. Because UCNPs were only seen in Kupffer cells at 24 hours after the injection (Fig. 5k,l) and there was no discrete uptake in the intestinal excretion from 24 hours after the injection (Fig. 2a). Also our speculation is supported by the previous knowledge that once NPs are phagocytosed by Kupffer cell, the NPs will be retained in the cells unless the NPs are broken down by intracellular process^19^.

Using NPs in human body is a very challenging goal. Even though large amount of the UCNPs was excreted in the present study, we could not exclude the possibility of long term adverse health effect by the remained UCNPs. Using the amount of the UCNPs as low as possible could be one way to further lower the possible toxicity^9^,^32^.

*In vivo* imaging using UCNPs is still quite challenging because the depth of tissue penetration is still limited. Optical imaging property of UCNPs was very helpful to confirm the excretion pattern in the present study, however to use the UCNPs based NPs for a generalized *in vivo* imaging agent, the NPs and imaging tool should be further optimized. Using UCNPs with more penetrable excitation light^33^ or applying further surface coating to lower quenching by water molecule^34^ could be ways to improve in vivo imaging ability of UCNPs.

In the present study, micelle encapsulated ^64^Cu-NOTA-UNCPs showed 75% of serum stability at 24 hours. 25% of instability in the human serum of the micelle encapsulated ^64^Cu-NOTA-UNCPs might be caused by protein adsorption to the NPs which could alter metabolic pathway of the NPs. However, according to PET image and biodistribution data in the present study, the NPs cleared from blood pool within 1 hour and observed serum stability of the micelle encapsulated ^64^Cu-NOTA-UCNPs was almost 100% until 4 hours. Thus we could assume that protein adsorption on the NPs would not significantly change the metabolic pathway of the micelle encapsulated ^64^Cu-NOTA-UCNPs.

In conclusion, we demonstrated feasibility of bimodal *in vivo* imaging characteristics of micelle encapsulated ^64^Cu-NOTA-UCNP and showed the substantial hepatobiliary excretion through *in vivo* microPET, ULI, and *ex vivo* biodistribution study. We also confirmed that our micelle encapsulation method worked and its product ^64^Cu-NOTA-UCNP distributed *in vivo* as integral entity which finally excreted by hepatobiliary routes. Thus, we propose that micelle encapsulated ^64^Cu-NOTA-UCNP exploiting its multiplexing capabiltiy, by adding further functional moiety, could be used for bimodal targeting agent for simultaneous ULI and PET study for variety of purposes, for example, radio-theranostic UCNP with multiplexed targeting ligands and chelator for therapeutic ^177^Lu.

## Materials and Methods

### Chemicals

YCl_3_∙6 H_2_O (99.9%), YbCl_3_∙6H_2_O (99.9%), ErCl_3_∙6H_2_O (99.9%), oleic acid (technical grade, 90%), 1-octadecene (technical grade, 90%) and NH_4_F (98.0%) were purchased from Aldrich. Sodium oleate was purchased from TCI. 1,2-distearoyl-sn-glycero-3-phosphoethanolamine-N-[methoxy(polyethyle-ne glycol)-2000] (mPEG-2000 PE) was purchased from Avanti Polar Lipids, Inc. All reagents were used as received.

### Synthesis of hexagonal phase NaYF_4_:Yb^3+^/Er^3+^ nanoparticles (UCNPs)

The hexagonal UCNPs were synthesized as described in a report by Li *et al.*^35^. Y-oleate, Yb-oleate and Er-oleate complexes were prepared by the methods reported previously^36^. Briefly, 0.78 mmol of Y-oleate, 0.2 mmol of Yb-oleate and 0.02 mmol of Er-oleate complexes were mixed with 10 mL of oleic acid and 15 mL of 1-octadecene in a 100 mL three-neck round bottom flask. And the reaction mixture was heated up to 100 °C under vacuum with stirring for 30 min to remove residual water and oxygen and then cooled the solution to room temperature. 10 mL of methanol solution containing 2.5 mmol of NaOH and 4 mmol of NH_4_F was slowly added into the reaction vessel under Ar. The reaction mixture was stirred for 30 min at 50 °C. The reaction mixture was heated to 100 °C under vacuum with stirring for 30 min to remove methanol. Then the reaction mixture was heated to 300 °C at a constant heating rate of 3.3 °C/min, and then kept at that temperature for 1 hour under Ar. The resulting solution was then cooled at room temperature. No further purification was needed and the UCNPs showed uniform size of 28 × 34 nm which was measured from TEM images using ImageJ software (NIH). Concentration and composition of UCNPs were analyzed by inductively coupled plasma mass spectrometer (ICP-MS, ELAN 6100, Perkin Elmer) to be 113.88 nM and Y:Yb:Er = 86.5:12.4:1.1 (mol%), respectively.

### Synthesis of chelating agent conjugated with stearyl chains (NOTA-C_18_)

1-(*p*-benzyl-1′,4′,7′-triazacyclononane-1′,4′,7′-triacetic acid)-3-octadecylthiourea (NOTA-C_18_) for ^64^Cu labeling was synthesized by conjugating 2-(*p*-isothiocyanatobenzyl)-1,4,7-triazacyclononane-1,4,7-triacetic acid (SCN-Bn-NOTA) and stearylamine. SCN-Bn-NOTA (20 mg, 0.04 mmol) was dissolved in CHCl_3_ (1 mL), and TEA (0.012 mL, 0.09 mmol) was added and stirred at room temperature. Stearylamine (14 mg, 0.05 mmol) was then added to the reaction mixture and stirred for 20 hours. The product was purified by the column chromatography on silica-gel (230–400 mesh) with CH_2_Cl_2_:MeOH (9:1) mixture as a eluent. The structure was confirmed by mass spectroscopy. Yield: 26 mg (72%). Mass spectrum (ESI^ + ^), (M^+^H^+^): 821.8. HRMS (M^+^H^+^): observed 720.4731, calculated 720.4734.

### Encapsulation of UCNPs with amphiphiles

The synthesis of water-soluble UCNPs was followed by the previous protocol^14^. Polysorbate 60 was the only commercially available (Sigma-Aldrich) amphiphile used in this study. 4% (v/v) polysorbate 60 solution in distilled water (1 mL) was added to NOTA-C_18_. The mixture was then heated to 80 °C at 20 min.

After the removal of hexane from UCNPs with inert gas, functionalized amphiphiles (2% [mol/mol] of polysorbate 60) were added. The mixture was sonicated for 80 min using VialTweeter at UIS250v at ~84.5 watt (Hielscher Ultrasonics GmbH, Germany). The reaction mixtures containing UCNPs were applied to a Sephacryl S-400 column (Sigma-Aldrich) (6.7 × 150 mm) and eluted with distilled water to remove unbound polysorbate 60 and other amphiphiles. Fractions (1 mL) were collected and concentrated by ultrafiltration (Amicon Ultracel, 100-kDa cutoff; Millipore).

### Size measurement by nanoparticle tracking analysis

The size distribution was checked by the nanoparticle tracking analysis (NTA) method using a NanoSight NS500 (Malvern, Grovewood road, UK) by using minor modification of manufacturer’s methods. Samples were diluted sufficiently for the contrast and minimal background level. The quick measurement mode was performed to find the optimal condition. Then, total 5 numbers of particle motion video were recorded automatically using standard measurement mode (temperature: 20.3 or 20.4 °C and viscosity: 0.99 to 1.0 cP). All other conditions were constant.

### ^64^Cu radiolabeling

^64^Cu was produced from cyclotron PETtrace 10 (GE healthcare, Sweden). The micelle encapsulated NOTA-UCNPs (32 pmol, 10 μL) was mixed with ^64^CuCl_2_ (5–6 mCi/0.1 mL) in the presence of 1 M sodium acetate buffer (pH 5.6, 0.3 mL). Then the reaction mixture was incubated for 10 min at 45 °C. The labeling yield was determined by ITLC-SG (1 × 10 cm strip) eluted with 0.1 M citric acid. The ITLC-SG strip was scanned using a TLC scanner. For the *in vivo* study, micelle encapsulated ^64^Cu-NOTA-UCNPs was concentrated using an Amicon filtration system (Millipore).

### Stability test

The stabilities of micelle encapsulated ^64^Cu-NOTA-UCNPs (0.37 MBq) in phosphate-buffered saline at room temperature and in human serum at 37 °C were determined by instant thin-layer chromatography-silica gel (ITLC-SG) plates (Pall Corp., U.S.A.) with 0.1 M citric acid at 0, 10, 30, 60 min and 2, 4, 8, 24 hours. The ITLC-SG strip was scanned using a TLC scanner (AR-2000, Bioscan, U.S.A.). Micelle encapsulated ^64^Cu-NOTA-UCNPs were stable for more than 24 hours in phosphate-buffered saline at room temperature (100%) and in human serum at 37 °C (75%) (Supplementary Fig. 2).

### *In vivo* microPET imaging of micelle encapsulated ^64^Cu-NOTA-UCNPs, ^64^Cu-NOTA and free ^64^Cu injected mice

Specific pathogen-free 6-week-old male BALB/c mice were obtained from SLC Inc. (Japan). All of the animal experiments were approved by Institutional Animal Care and Use Committee of the Clinical Research Institute at Seoul National University Hospital and performed in accordance with the guideline from the institute. In addition, the National Research Council guidelines for the care and use of laboratory animals (revised 1996) were observed throughout.

Using micelle encapsulated ^64^Cu-NOTA-UCNPs and animal PET (GENISYS, Sofie Biosciences, Culver City, CA), the biodistribution study in BALB/c mice was evaluated. After ^64^Cu -NOTA-UCNPs were injected to the tail vein of mice (weight = 24.1 ± 0.5 g, dose = 40 ± 0.6 μCi/50 μL), animal PET was performed to obtain serial time point images (0.25, 1, 2, 4, 8, and 24 hours, n = 5/0.25, 1, 2, 4, 8, 24, 48 and 72 hours, n = 4). Images were acquired for 15 minutes until 24 hours, and acquired for 30 and 60 minutes at 48 hours, and 72 hours respectively. For the comparison study of free ^64^Cu (n = 3), ^64^Cu-NOTA-C_18_ (n = 3) and micelle encapsulated ^64^Cu-NOTA-UCNPs (n = 9), BALB/c mice were prepared. Dose of ^64^Cu was 35 μCi/50 μL (19.9 g) and dose ^64^Cu-NOTA-C_18_ was 40 μCi/50 μL (18.6 g) by tail vein injection before PET image acquisition. Dynamic whole-body PET images were obtained during 15 min in 20 frames (10 × 60 s). The images were obtained by 3-dimensional Fourier rebinning using a 2-dimensional ordered-subsets expectation maximization reconstruction algorithm with scatter, decay, and attenuation correction from raw framed sonograms. In each PET image, 3-dimensional regions of interest were drawn over major organs on whole-body axial images. Mean standardized uptake values (meanSUV) were obtained using PMOD software from reconstructed data. Animal PET was performed serially at 30 min, after injection under isoflurane inhalation anesthesia. After acquisition of 24 hours delayed PET image, mice were sacrificed. All liver and spleen were fixed in formaline solution. Feces and urine of mice after injection of micelle encapsulated ^64^Cu-NOTA-UCNPs were evaluated by PET image to confirm the excretion of micelle encapsulated ^64^Cu-NOTA-UCNPs. The biodistribution and microPET experiments were performed in Seoul National University Hospital, which is fully accredited by AAALAC International (2007, Association for Assessment and Accreditation of Laboratory Animal Care International).

### Biodistribution study using micelle encapsulated ^64^Cu-NOTA-UCNPs and *ex vivo* method

All mice used were male BALB/c mouse (4 ~ 5 weeks old) obtained from the breeding facility of the Seoul National University Hospital Biomedical Research Institute. Micelle encapsulated ^64^Cu-NOTA-UCNPs were injected into a male BALB/c mouse via the tail vein (weight = 20.1 g, dose = 1 μCi/100 μL). The injected mice were sacrificed in serial time points (1, 4, 8, 24, and 72 hours, 3 mice for collection of feces and urine, n = 3, respectively, total n = 18) of post-injection. Blood, liver, muscle, kidney, lung, heart, small intestine, large intestine, bone and other organs were then excised, blotted and weighed, and then ^64^Cu radioactivity of each organ was counted by a gamma scintillation counter (DREAM r-10, Shinjin Medics Inc., South Korea). The results are expressed as percentages of injected doses per gram of tissue (%ID/g).

### Preparation of intraluminal feces, feces and urine smear section for upconverting luminescence imaging

Whole large and small intestine of BALB/c nude mouse after 8 hours injection of UCNPs was cut about 3 cm size and each resected intestine was put on the slide glass. For reducing penetration scattering of photoluminescence, we dissected intestinal lumen vertically in every 3 cm size intestine. The specimens were extended on the slide glass and the other glass was compressed. Feces were collected for the serial time point. Individual feces was compressed and enlarged by the slide glass for the better optical image. Spotty urine from mice after 30 min and 1 hour injection of UCNPs was collected and normal saline only was mixed for the slide glass smear. Photoluminescence imaging of feces and urine were obtained, respectively.

### Upconverting luminescence spectra of UCNPs

In-house made optical imaging apparatus for *in vivo* UCNP imaging was used. The UCNP solutions were excited by 980-nm CW laser (SDL-980LM-500 T, Shanghai Dream Lasers Technology) and the emission was collected at right angle by an optical fiber and detected by a CCD camera (PIXIS 400BR, Princeton Instruments) attached to a monochromator (HoloSpec f/1.8, Kaiser Optical Systems). It was composed of an inverted microscope (TE2000-U, Nikon), an NIR (980 nm) diode laser (P161-600-980 A, EM4), and an electron multiplying CCD (EMCCD) camera (DV897DCS-BV, iXon, Andor Technology). The output of the 980 nm laser was reflected by a short-pass dichroic beam splitter (725dcspxr, Chroma Technology) and directed to the microscope objective (Plan Apo VC, 60X, NA 1.40, oil immersion, Nikon). The beam was focused on the back focal plane of the objective by a planoconvex lens (focal length 400 mm) resulting in the illumination area with diameter of ca. 60 mm. The typical power density of illumination was 300 W/cm^2^ on the sample surface. The emission from the UCNPs in the visible range was collected by the same objective, passed through the dichroic beam splitter and a short-pass emission filter (ET700sp-2p, Chroma Technology), magnified further by a set of achromatic lenses outside the microscope, and finally imaged by the EMCCD camera. The transmission range of the emission filter (400–700 nm) covers both the green (centered at 525 and 545 nm) and red (centered at 657 nm) emission bands from UCNPs. We also obtained bright field images of feces and urine samples using the same EMCCD camera and a lamp as the light source.

### Acquisition of upconversion luminescence imaging

The *in vivo* upconversion luminescence imaging in BALB/c nude mice was performed after tail vein injection of UCNPs (weight = 24.1 g, dose = 0.12 mg/300 μL, total n = 7). Injection dose was escalated from 40 ug/mouse, and starting at the dose of 158 ug/mouse, we could obtain sufficient signals for *in vivo* luminescent imaging which is 13 times higher amount than that for the dose of PET imaging (12 μg/mouse). Before the image acquisition, peritoneal anesthesia in mice was performed. *In vivo* photoluminescence imaging was obtained as serial time points (1, 2, 4, 8, and 24 hours). For *in situ* image, nude mouse skin and peritoneum was exfoliated. After sacrifice, *ex vivo* image was performed. All images were acquired under the same experimental condition (980 nm laser power = 21 W (30 A), ~300 mW/cm^2^, EMCCD Gain = 250, exposure time = 10 s).

### Acquisition of TEM

TEM images were taken at an acceleration voltage of 80 keV (JEM-1400; Jeol). To obtain negative-stain TEM images of UCNPs and micelle encapsulated UCNPs, UCNPs solutions were dropped onto a Formvar carbon-coated copper grid (SPI-Chem) and stained with saturated uranyl acetate solution. To observe hepatobiliary excretion, liver specimen was carefully selected. Liver tissue from micelle encapsulated ^64^Cu-NOTA-UCNPs injected mice was chopped and multiple pieces were selected including 5 regions of both lobes of liver, 1 hour (n = 3) and 24 hours after injection (n = 4).

## Additional Information

**How to cite this article**: Jung Seo, H. *et al.* Rapid Hepatobiliary Excretion of Micelle-Encapsulated/ Radiolabeled Upconverting Nanoparticles as an Integrated Form. *Sci. Rep.* **5**, 15685; doi: 10.1038/srep15685 (2015).

## Supplementary Material

Supplementary Information
