# Supplementary material for: Rapid Hepatobiliary Excretion of Micelle-Encapsulated/Radiolabeled Upconverting Nanoparticles as an Integrated Form
Source: Sci Rep. 2015 Oct 23;5:15685. doi: 10.1038/srep15685 (PMC4616227; doi:10.1038/srep15685)
Supplement: Supplementary Information [file srep15685-s1.doc]

**Supplementary figures**

**Rapid Hepatobiliary Excretion of Micelle-Encapsulated/ Radiolabeled Upconverting Nanoparticles as an Integrated Form**

Hyo Jung Seo^1,3†^, Sang Hwan Nam^4†^, Hyung-Jun Im^1,3†^, Ji-yong Park^3^, Ji Youn Lee^3^, Byeongjun Yoo^5,6^, Yun-Sang Lee^1,3^, Jae Min Jeong^3^, Taeghwan Hyeon^5,6^, Ji Who Kim^3^, Jae Sung Lee^3^, In-Jin Jang^2^, Joo-Youn Cho^2^, Do Won Hwang^1,3, *^, Yung Doug Suh^4,7,*^, and Dong Soo Lee^1,3,*^

**Affiliation:**

^1^Department of Molecular Medicine and Biopharmaceutical Sciences, Graduate School of Convergence Science and Technology, Seoul National University, Seoul, Korea

^2^Department of Clinical Pharmacology and Therapeutics, Seoul National University College of Medicine, Seoul, Korea

^3^Department of Nuclear medicine, Seoul National University College of Medicine, Seoul, Korea

^4^Laboratory for Advanced Molecular Probing (LAMP), Research Center for Convergence Nanotechnology, Korea Research Institute of Chemical Technology, Daejeon, Korea

^5^Center for Nanoparticle Research, Institute for Basic Science (IBS), Seoul, Korea

^6^School of Chemical and Biological Engineering, Seoul National University, Seoul, Korea.

^7^School of Chemical Engineering, Sungkyunkwan University, Suwon, Korea

[^†^] HJ Seo, SH Nam, HJ Im are equally contributed to this work as a first author.

[^§^] DW Hwang, YD Suh and DS Lee are equally contributed to this work as a corresponding author.


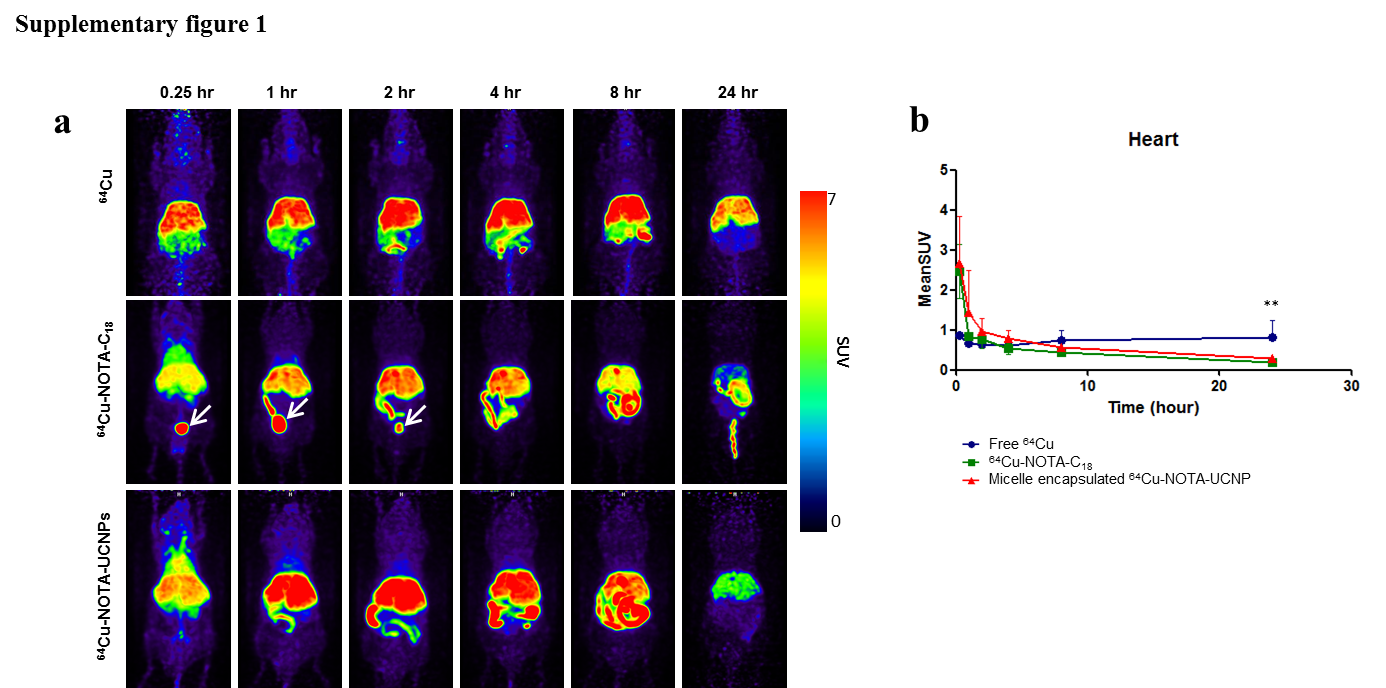


**Supplementary Figure 1.** Comparison of *in vivo* PET images using free ^64^Cu, ^64^Cu-NOTA-C_18_, and micelle encapsulated ^64^Cu-NOTA–UCNPs. **a**, PET images of mice at different time points after intravenous injection of free ^64^Cu, ^64^Cu-NOTA-C_18_ and micelle encapsulated ^64^Cu-NOTA-UCNPs, respectively. Arrow indicates bladder. **b**, Uptake in heart was higher in free ^64^Cu than ^64^Cu-NOTA-C_18_ and micelle encapsulated ^64^Cu-NOTA–UCNPs injected mice at 24 hour delay image (One-way analysis of variance (ANOVA) followed by post hoc test, **: P < 0.01).


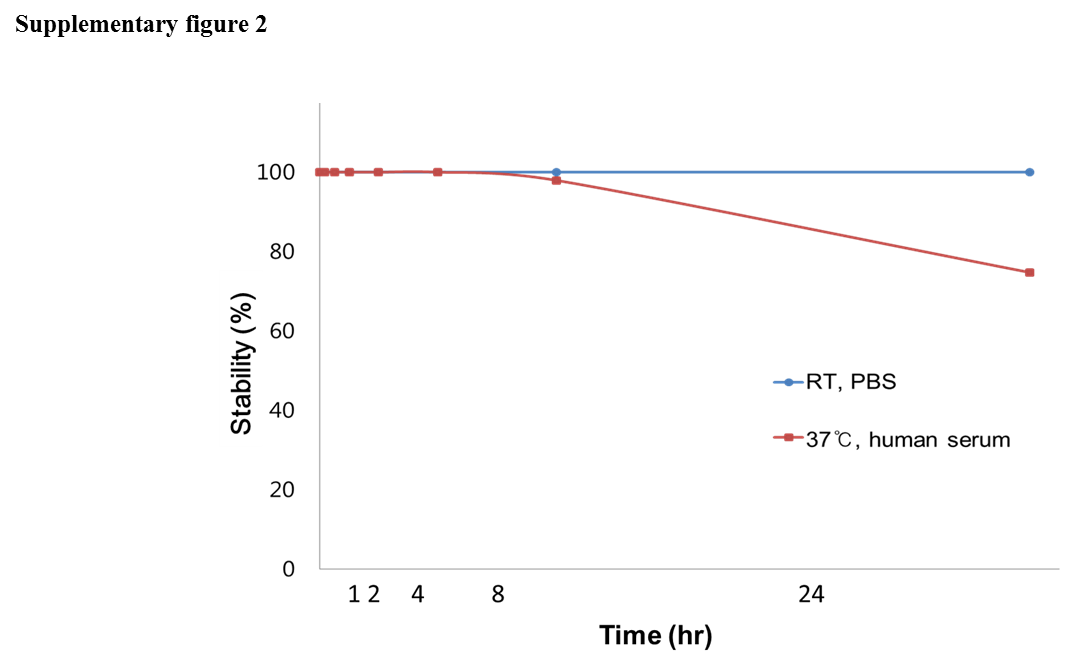


**Supplementary Figure 2.** Stability test of micelle encapsulated ^64^Cu-NOTA-UCNPs in PBS at room temperature and in human serum at 37 ˚C at different time points (0, 10, 30, 60 min and 2, 4, 8, 24 hours). Micelle encapsulated ^64^Cu-NOTA-UCNPs in PBS at room temperature were stable showing 100% labeling efficiency at 24 hrs. micelle encapsulated ^64^Cu-NOTA-UCNPs in human serum at 37˚C were stable showing 98% labeling efficiency till 8 hours, and 75% stability at 24 hours.
